# Suicide rates among patients subject to community treatment orders in England during 2009–2018

**DOI:** 10.1192/bjo.2021.1021

**Published:** 2021-10-01

**Authors:** Isabelle M. Hunt, Roger T. Webb, Pauline Turnbull, Jane Graney, Saied Ibrahim, Jenny Shaw, Nav Kapur, Louis Appleby

**Affiliations:** National Confidential Inquiry into Suicide and Safety in Mental Health, Centre for Mental Health and Safety, Faculty of Biology, Medicine and Health, University of Manchester, UK; Manchester Academic Health Sciences Centre, University of Manchester, UK; and NIHR Greater Manchester Patient Safety Translational Research Centre, University of Manchester, UK; National Confidential Inquiry into Suicide and Safety in Mental Health, Centre for Mental Health and Safety, Faculty of Biology, Medicine and Health, University of Manchester, UK; National Confidential Inquiry into Suicide and Mental Health, Centre for Mental Health and Safety, Faculty of Biology, Medicine and Health, University of Manchester, UK; National Confidential Inquiry into Suicide and Safety in Mental Health, Centre for Mental Health and Safety, Faculty of Biology, Medicine and Health, University of Manchester, UK; National Confidential Inquiry into Suicide and Safety in Mental Health, Centre for Mental Health and Safety, Faculty of Biology, Medicine and Health, University of Manchester, UK; NIHR Greater Manchester Patient Safety Translational Research Centre, University of Manchester, UK; and Greater Manchester Mental Health NHS Foundation Trust, UK; National Confidential Inquiry into Suicide and Safety in Mental Health, Centre for Mental Health and Safety, Faculty of Biology, Medicine and Health, University of Manchester, UK

**Keywords:** Community treatment orders, suicide, psychiatric patients, mental health, mental health act

## Abstract

**Background:**

Community treatment orders (CTOs) enable patients to be treated in the community rather than under detention in hospital. Population-based studies of suicide among patients subject to a CTO are scarce.

**Aims:**

To compare suicide rates among patients subject to a CTO with all discharged psychiatric patients and those detained for treatment but not subject to a CTO at discharge (‘CTO-eligible’ patients).

**Method:**

From a national case series of patients who died by suicide within 12 months of contact with mental health services in England during 2009–2018, we estimated average annual suicide rates for all discharged patients, those on a CTO at the time of suicide, those ever treated under a CTO and CTO-eligible patients.

**Results:**

Suicide rates for patients on a CTO at the time of suicide (191.3 per 100 000 patients) were lower than all discharged patients (482.3 per 100 000 discharges). Suicide rates were similar in those ever treated under a CTO (350.1 per 100 000 CTOs issued) and in CTO-eligible patients (382.9 per 100 000 discharges). Suicide rates within 12 months of discharge were higher in persons ever under a CTO (205.1 per 100 000 CTOs issued) than CTO-eligible patients (161.5 per 100 000 discharges), but this difference was reversed for rates after 12 months of discharge (153.2 per 100 000 CTOs issued *v*. 223.4 per 100 000 discharges).

**Conclusions:**

CTOs may be effective in reducing suicide risk. The relative benefits of CTOs and intensive aftercare may be time-dependent, with the benefit of a CTO being less before 12 months after discharge but greater thereafter. CTO utilisation requires a careful balancing of patient safety versus autonomy.

## Background

Suicide represents a global public health problem, with over 700 000 people dying by suicide each year.^1^ According to the most recent data in England and Wales, in 2019, there were 5691 suicides registered, representing 11.0 deaths per 100 000 population.^2^ Mental illness is strongly associated with an increased suicide risk^3^ and all member states of the World Health Organization (WHO) have committed to improving mental health and reaching a global target of reducing suicide rates by a third by 2030.^4^ Identifying high-risk groups is a key preventative goal, and people under the care of mental health services, including in-patients, have been identified as a priority in England's National Suicide Prevention Strategy.^5^ For those with severe mental illness with impaired decision-making capacity, the use of compulsory admission and detainment for treatment under the Mental Health Act 1983 (MHA; amended 2007) is considered where there is a potential risk of harm to the patient or others. Two common detaining sections of the MHA are Section 3 (admission for treatment) and Section 37 (hospital order for those convicted of a criminal offence).

The introduction of community treatment orders (CTOs) in England and Wales in 2008 provided the option for people detained under the MHA to be treated in the community rather than in hospital. Their purpose was to reduce levels of readmission and provide supervised treatment in a less restrictive environment, while maintaining close clinical contact. Two mandatory conditions attached to a CTO involve the availability of the patient for assessment at the time of CTO renewal, and agreement to be seen by a second psychiatrist if the individual is too unwell to consent to receiving drug treatment themselves. Discretionary conditions can also be applied with the patient's agreement, typically prescribed treatment adherence and service engagement. Although treatment is not coercively imposed on patients in the community, those who show deterioration in mental health may be recalled back to hospital for assessment and medication. Qualitative studies have indicated that patients benefit from closer involvement in their care planning under a CTO,^6,7^ and family caregivers have viewed CTOs as a ‘safety net’, welcoming the option of recall at times of relapse.^8,9^ However, the coercive nature of supervised community treatment has caused much debate, with critics viewing CTOs as punitive, restricting civil liberties^10^ and compromising trust in the patient–psychiatrist relationship.^11^ A recent systematic review and meta-analysis found inconsistent evidence that compulsory community treatment improved patient outcomes.^12^ It also confirmed findings from the only randomised controlled trial of CTOs in England, the Oxford Community Treatment Order Evaluation Trial (OCTET), that CTOs did not reduce rates of readmission.^13,14^ Other studies in England have reported patients on a CTO had more hospital readmissions and spent longer in in-patient care compared with those not on a CTO.^15,16^ Despite the sparse evidence on whether CTOs reduce bed use and improve treatment response, they are used extensively in many countries and are often initiated to potentially reduce the risk of self-harm and suicide after discharge.

## CTOs and suicide

The National Confidential Inquiry into Suicide and Safety in Mental Health (NCISH)^17^ found 126 (1%) deaths by suicide among patients with recent mental health service contact were subjected to a CTO during 2009–2017, two-thirds of whom were on a CTO when they died. Of note, a third of patients subject to a CTO who died within 3 months of discharge were preceded by treatment non-adherence and missed service contact – two key elements of CTO provision. The evidence base for the effectiveness of CTOs in reducing suicide is limited: one UK observational controlled study found no association between CTOs and suicide risk among patients with schizophrenia,^18^ whereas a USA study found that mandated community treatment reduced suicide risk.^19^ Further knowledge is needed to enhance our understanding of the clinical profile of patients who are placed on a CTO who subsequently die by suicide. We utilised a national case series to examine suicide risk in patients who had been treated under a CTO. Our objectives were to calculate suicide rates among patients under a CTO, and to compare rates and features of patients subject to a CTO with those who were not under CTO but who were eligible to be so; i.e. they had been detained for treatment under Section 3 or 37 of the MHA at their last admission. For this comparison, we examined suicides among patients who had ever been under CTO after hospital discharge, to take into account the possibility that the period at risk for patients dying when under a CTO may have been shorter than the equivalent period for those who were eligible for a CTO. We hypothesised that suicide risk would be highest among patients subject to a CTO (on the grounds that management without legal restriction had been viewed by clinicians as inadequate), followed by CTO-eligible patients and then all other patients discharged from in-patient psychiatric care.

## Method

### Study data-set

We used data from the NCISH. A detailed description of NCISH methodology is described elsewhere.^20^ In brief, there are three stages to data collection. First, NCISH is notified from the Office for National Statistics (http://wwww.ons.gov.uk) of all unnatural deaths in England with a coronial verdict of suicide or of undetermined intent (‘open’ verdict). Second, details of the deceased are sent to mental health services in the individual's residential district to identify those who had been in service contact in the 12 months before death (‘patient suicides’). Third, sociodemographic and clinical data on patient suicides are collected via a questionnaire completed by the responsible clinician. The questionnaire collects detailed information on patients who died by suicide in the community, including whether the patient was on a CTO at the time of their last discharge or death, and the length of time spent under a CTO (<6 months, between 6 months and 1 year, and ≥1 year). In addition, the nature of patients’ previous admissions (voluntary, detained for assessment or treatment, under the power of recall of a CTO) were recorded.

Deaths that were assigned an open verdict were included in the study, as is conventional in suicide research in the UK,^21^ and are hereon referred to as ‘suicides’ in this paper. We selected all patients who died by suicide during years 2009 and 2018, inclusive.

### Denominator data

For overall suicide rates among discharged patients, we applied the number of discharges as the denominator, obtained from the Mental Health Services Data Set (MHSDS) via NHS Digital.^22^ This national data-set holds patient-level data on all individuals in contact with in-patient, out-patient and community mental health services. To calculate suicide rates among patients who were ever subject to a CTO, we applied the number of open CTOs for the years 2009/10 to 2018/19 as the denominator. We also calculated an overall suicide rate among patients who were on a CTO at the time of death, using the denominator figures on 31 March to give a typical rate on any one day (MHSDS data were available from 2010 onward). Suicide rates among CTO-eligible patients (i.e. those detained for treatment under Section 3 or Section 37 of the MHA, but who were discharged to voluntary community care) were calculated per 100 000 admissions under Section 3 or Section 37 (excluding discharges from a CTO), with MHSDS data available for the years 2011–2018. Denominator data to calculate these rates included conversions to a Section 3 or Section 37 for those who had been voluntarily admitted. The numbers of admissions, discharges and CTOs issued were available by financial year initially; we then converted them to being by calendar year, for consistency with the numerators.

### Statistical analysis

We first calculated suicide rates (and their 95% confidence intervals) across the entire observation period for all patients discharged from psychiatric care, as a baseline against which suicide rates in the subgroups of interest could be compared. Suicide rates among patients ever subject to a CTO were calculated by the total number of CTOs issued; suicide rates among patients on a CTO at the time of death were calculated by the total number of patients on a CTO on the 31 March of each year. Suicide rates among CTO-eligible patients were compared with those for persons ever treated under a CTO, stratified according to whether they had died within or after 12 months of discharge. Calendar year was fitted as a continuous variable in a Poisson regression model, to test for linear temporal trends in the suicide rate.^23^ Poisson models were tested for evidence of significant overdispersion, using the likelihood ratio test of *a* = 0 that is generated by the negative binomial model. Where tests indicated inadequate goodness of fit, the parameters from the negative binomial model were reported instead of those from the Poisson model.^23^ Because of small numerator values, we collapsed years into categories of 2011–2014 and 2015–2018 and compared rates between patients subject to a CTO and CTO-eligible patients via incidence rate ratios (IRRs) and their exact 95% confidence intervals. Comparison of patient characteristics between those on a CTO and all other patients (i.e. those not treated under a CTO) were conducted with chi-squared tests, with statistical significance indicated at the two-sided 5% level.

Over the study's observation period, the overall response rate for questionnaires was 95%, but was lower during calendar years 2016 (94%), 2017 (90%) and 2018 (71%) because of the time associated with legal processes and data collection. We therefore estimated the number of suicides in 2016–2018, based on the expected final return of questionnaires for the previous 7 years (2009–2015), to enable comparable numerator data when assessing rates across the study period. Descriptive statistics of patient characteristics were carried out on actual cases. All statistical analysis was performed with Stata software version 15.1 for Windows.^24^ Ethical approval was obtained from the North West – Greater Manchester South Research Ethics Committee (reference: ERP/96/136). NCISH also has Section 251 approval under the NHS Act 2006 (reference: PIAG 4-08(d)/2003).

## Results

Over the study period 2009–2018, there were 12 771 suicides by people who had been in contact with mental health services in the 12 months before they died. Of these, 11 921 (93%) were patients treated in the community, and 143 were treated under a CTO at their last discharge (referred to as ‘ever subject to a CTO’ herein); 3.9% of all community patient suicides with a previous admission, or 1.2% of all community patient suicides. This represents a mean of 14 suicide deaths per year in those ever subject to a CTO. At date of death, 93 patients were still on a CTO and 47 had had their CTO revoked; in the remaining three cases, CTO status at date of death by suicide was unknown. There were 483 CTO-eligible patients during the observation period, i.e. those who had been detained for treatment but were not under a CTO at discharge. This group represented 7.4% of all community patient suicides with a previous admission, or 4.1% of all community patient suicides. Suicide rates among CTO-eligible patients were calculated for those who died during 2011–2018 (*n* = 377) as denominator data were available for that period only.

### Characteristics of patient suicides ever treated under a CTO

The median age of patients ever subject to a CTO (*N* = 137) was 44 years (interquartile range 16 years) and 85 out of 137 (62%) were male. Over a quarter (39 out of 137, 28%) were from a minority ethnic group, proportionally more than other patients (832 out of 10 599, 8%; *P* < 0.001). The suicide method profile of the CTO group differed from that of other patient suicides, with a higher proportion jumping from a height or in front of a moving vehicle (46 out of 136, 34% *v*. 1492 out of 10 851, 14%; *P* < 0.001) and a lower proportion of self-hangings/strangulations (42 out of 136, 31% *v*. 4974 out of 10 851, 46%; *P* = 0.001). The most common primary psychiatric diagnosis was schizophrenia and other delusional disorders (108 out of 137, 79% *v*. 1536 out of 10 688, 14% of other patients; *P* < 0.001). Patients subject to a CTO more often had coexisting drug dependence or misuse (37 out of 137, 27% *v*. 1075 out of 10 633, 10%; *P* < 0.001).

### Suicide rates

Between 2010 and 2018, the overall suicide rate among patients who were under a CTO at date of death was 191.3 per 100 000 CTO patients on the 31 March (Table 1). There was no overall linear temporal trend in rates across the study period (linear trend; 1 d.f. = 1.08; *P* = 0.30; see Supplementary Fig. 1 available at https://doi.org/10.1192/bjo.2021.1021). Suicide rates among patients under a CTO were lower compared with all patients discharged from in-patient care at 482.3 per 100 000 discharges (IRR 0.40; 95% CI 0.32–0.49; *P* < 0.001) (Table 1). There was a fall in the number (linear trend; 1 d.f. = 8.5; *P* = 0.004) and rate (linear trend; 1 d.f. = 4.22; *P* = 0.04) of suicide among discharged patients across the observation period (Supplementary Fig. 1).

**Table 1:** Suicide rates among all discharged patients and among patients on a CTO when they died

	All discharged patients			Patients on a CTO at date of death			Relative risks: IRRs		
Period of observation, years	*n*	Rate^a^	95% CI	*n*	Rate^b^	95% CI	IRR	95% CI	*P*-value
2009–2013	3052	515.4	497.4–534.0	37^c^	210.3	148.1–289.7	0.41	0.29–0.56	<0.001
2014–2018	2641	449.0	432.1–466.4	48	178.8	131.9–237.0	0.40	0.29–0.53	<0.001
Total	5693	482.3	469.9–495.0	85	191.3	152.8–236.4	0.40	0.32–0.49	<0.001

CTO, community treatment order; IRR, incidence rate ratio.

a. Rate per 100 000 discharges from in-patient care.

b. Rate per 100 000 patients subject to a CTO on the 31 March of each year.

c. Data shown for 2010–2013 as denominator incomplete in 2009, therefore the total does not total 93 as reported in the main text.

Table 2 shows the numbers and rates of suicide among patients ever subject to a CTO and among CTO-eligible patients (i.e. those detained for treatment but were not under a CTO at discharge). Although the number of CTOs issued increased across the observation period, the number and rate of suicide fluctuated, and there was no evidence of linear temporal trend (1 d.f. = 0.07; *P* = 0.79). There was, however, a significant increase in the rate of suicide in CTO-eligible patients between 2011 and 2018 (linear trend; 1 d.f. = 11.38; *P* = 0.001).

**Table 2:** Suicide rates among patients ever placed on a CTO and among CTO-eligible patients

	Ever placed on a CTO			CTO-eligible patients			Relative risks: IRRs		
Period of observation, years	*n*	Rate^a^	95% CI	*n*	Rate^b^	95% CI	IRR	95% CI	*P*-value
2011–2014	74	418.5	328.7–525.1	172	325.9	279.1–378.4	0.78	0.67–0.90	0.001
2015–2018	54	286.0	214.9–373.0	205	448.6	389.4–514.3	1.57	1.36–1.80	<0.001
Total	128	350.1	292.1–416.1	377	382.9	345.3–423.5	1.09	0.99–1.21	0.09

CTO, community treatment order; IRR, incidence rate ratio.

a. Rate per 100 000 CTOs issued.

b. Rate per 100 000 discharges.

Annual suicide rates were significantly lower among patients on a CTO at the time of death (Table 1) compared with those ever subject to a CTO (350.1 per 100 000 CTOs issued; IRR 0.55; 95% CI 0.44–0.68; *P* < 0.001) and CTO-eligible patients (382.9 per 100 000 discharges; IRR 0.50; 95% CI 0.40–0.62; *P* < 0.001)) (Table 2). The suicide rate for CTO-eligible patients was slightly higher than for those ever treated under a CTO, but this difference did not reach statistical significance (IRR 1.09; 95% CI 0.99–1.21; *P* = 0.09). However, the rate among CTO-eligible patients who died within 12 months of discharge was lower than for patients ever subject to a CTO who died within 12 months of discharge (161.5 *v*. 205.1; *P* = 0.045; Table 3). The opposite was found when comparing suicide rates after 12 months of discharge (Table 4), when rates in patients ever subject to a CTO were lower than among patients who were CTO-eligible (153.2 *v*. 223.4; *P* = 0.003).

**Table 3:** Suicide rates among patients ever placed on a CTO and among CTO-eligible patients who died within 12 months of discharge

	Ever placed on a CTO			CTO-eligible patients			Relative risks: IRRs		
Period of observation, years	*n*	Rate^a^	95% CI	*n*	Rate^b^	95% CI	IRR	95% CI	*P*-value
2011–2014	48	271.5	200.2–359.7	72	136.4	106.8–171.8	1.99	1.47–2.64	<0.001
2015–2018	27	143.0	94.3–208.0	87	190.4	152.5–234.8	0.75	0.49–1.09	0.13
Total	75	205.1	161.4–257.1	159	161.5	137.4–188.6	1.27	1.00–1.59	0.045

CTO, community treatment order; IRR, incidence rate ratio.

a. Rate per 100 000 CTOs issued.

b. Rate per 100 000 discharges.

**Table 4:** Suicide rates among patients ever placed on a CTO and among CTO-eligible patients who died more than 12 months after discharge

	Ever placed on a CTO			CTO-eligible patients			Relative risks: IRRs		
Period of observation, years	*n*	Rate^a^	95% CI	*n*	Rate^b^	95% CI	IRR	95% CI	*P*-value
2011–2014	27	152.7	100.7–222.1	100	189.5	154.2–230.4	0.81	0.53–1.17	0.26
2015–2018	29	153.6	102.9–220.5	120	262.6	217.8–313.9	0.58	0.39–0.84	0.002
Total	56	153.2	115.7–198.8	220	223.4	194.9–254.9	0.69	0.52–0.89	0.003

CTO, community treatment order; IRR, incidence rate ratio.

a. Rate per 100 000 CTOs issued.

b. Rate per 100 000 discharges.

## Discussion

Our results suggest that suicide under CTO is a rare event, accounting for around 1% of all psychiatric patient suicides that occur in the community in England, for an average of 14 deaths per year nationally. We found a lower suicide rate among patients who were under a CTO when they died, compared with the rate among all discharged patients. Suicide rates were significantly lower for patients under a CTO at death compared with both the rate in patients ever subject to a CTO and in CTO-eligible patients. Overall, the suicide rate among patients ever treated under a CTO was not significantly different from the rate among patients who were CTO-eligible. We also found a difference in rates in relation to time under a CTO, with higher suicide rates within 12 months of discharge among all patients subject to a CTO compared with those who were CTO-eligible. However, after 12 months of discharge, this difference reversed, with the suicide rate among patients subject to a CTO becoming significantly lower than the rate among CTO-eligible patients.

It is well-established that suicide risk is greatly elevated after discharge from in-patient psychiatric care.^25,26^ However, both patients under a CTO and those eligible for a CTO appear to be at lower suicide risk compared with all discharged patients. There are two reasons to consider that our findings may suggest a benefit from CTOs specifically. First, the CTO-eligible patients are likely to have been at lower risk than the CTO group, hence the decision not to treat under a CTO, and the comparison of suicide rates should take into account this possible difference in risk. Second, the lower suicide rate in those under a current CTO suggests that an active CTO confers most protection. Our finding of a higher suicide rate among patients subject to a CTO within 12 months of discharge, and a lower rate more than a year after discharge, may be an indication that clinical stability takes longer to establish for patients subject to a CTO and that the main benefit is seen long term. Although there may be a causal effect that is attributable to improved care, alternative explanations are also possible, such as a longer period of pre-discharge stability in patients preparing for CTO, or selection of patients whose illnesses are more responsive to treatment.

Lower all-cause mortality rates have been previously reported in patients subject to a CTO compared with control patients, with closer community follow-up and opportunity for early identification of physical health problems cited as possible explanations.^15,27^ Such findings provide support for the effectiveness of CTOs in providing intensive case management in the community. Other studies have also suggested that patients under CTO have better engagement with community teams and sustained contact with services compared with non-CTO groups,^28,29^ and published evidence has shown that optimising continuity of care for vulnerable patients is effective in reducing suicide risk.^30^

Our findings concur with those reported from previous studies, which showed that patients under a CTO have more severe mental illness and are younger compared with those discharged without a CTO.^15,29^ Over a quarter of the patients subject to a CTO who died by suicide in our study belonged to an ethnic minority group. An overrepresentation of certain ethnic minority groups under compulsory treatment has previously been reported,^31^ yet controlled observational studies have not found ethnicity to be associated with CTO receipt, after adjusting for associated demographic or clinical factors.^15,29^ Considering the known relationship between ethnicity and psychotic disorders,^32^ it may be that the higher proportion of minority ethnic patients under a CTO reflects the greater prevalence of schizophrenia among the patients subject to a CTO within the NCISH case series. In line with other studies,^29,33^ we also found that comorbid substance misuse was more prevalent among patients subject to a CTO, highlighting the more complex clinical profile of these patients.

### Strengths and limitations

This national study was based on robust case ascertainment and, to our knowledge, was the first to examine suicide rates among patients under a CTO. The findings should, however, be interpreted in light of certain limitations. First, the observational nature of the study precluded elucidation of causal associations. Second, we did not have specific information on risk assessments and the decision-making process for patients on a CTO. However, as risk is fundamental to the use of MHA powers, it seems likely that patients subject to its provisions are perceived as being higher risk than those who are not, whether as a result of clinical presentation or non-clinical risk factors. We also cannot establish whether specific interventions to reduce suicide risk were implemented with the patients subject to a CTO, thereby explaining the lower suicide rates. How clinician- or patient-related factors influence a CTO outcome is worthy of further study. Third, the retrospective data collection from clinicians was based on their knowledge of the patient, clinical judgement and case records, as opposed to standardised assessments. However, the reliability and validity of NCISH questionnaire data have been shown to be good, and the NCISH consistently achieves high response rates.^20,34^ Fourth, in patients subject to a CTO, the number of suicides that occurred across the study's observation period indicated an unstable pattern (most likely because of the small number of suicides that occurred each year), and our projected figures for the most recent years may be over- or underestimates. Fifth, denominator data were obtained from the MHSDS and therefore were not specifically collected for the purpose of conducting this study. We were therefore unable to stratify the results by age, gender or ethnicity, or estimate suicide rates by duration of care under CTO. Furthermore, some of the aggregate denominators that we applied to calculate rates may have contained multiple episodes; for instance, if more than one CTO had been issued or there were multiple discharges during a year, this may have slightly reduced the accuracy of these calculated rates. Nonetheless, the MHSDS represents the most complete source of national denominator data on mental health activity that is available for academic research in England. Finally, the findings were based on patients in England only and may not be generalisable to the devolved nations of the UK, or to other countries where CTOs are applied. They may also not be applicable to individual healthcare providers where the use of CTOs varies widely.^29^ Part of this variation may lie in differences in case-loads and procedures between healthcare settings, but may also reflect differing experiences of clinicians with regard to CTOs,^35^ which warrants further investigation.

In conclusion, the most important test of CTOs is whether they improve patient safety and lower suicide risk. This is difficult to accomplish via randomised controlled trials for practical and ethical issues, and therefore we are left with conducting prospective observational follow-up studies to further our understanding of the potentially beneficial effect of CTOs. Overall, the findings generated from the current study contribute to the debate as to whether CTOs are effective in enhancing patient safety. The UK Government's 2018 Independent Review of the Mental Health Act 1983^36^ considered the implications if CTOs were replaced or reformed. This review concluded that CTOs should remain for the time being, but be reduced in number, whereas their application criteria should be tightened. We have shown evidence of the beneficial effect of CTOs in potentially reducing suicide risk, but further monitoring is needed to determine whether the rates continue to remain lower than among patients who are treated voluntarily. CTOs remain a controversial treatment option and clinicians making judgements about the use of CTOs have to consider not only risk, but factors such as patient autonomy, long-term adherence and potential stigma.

## Data Availability

All authors had full access to all of the data in the study and take responsibility for the integrity of the data and the accuracy of data analysis; access to the data is currently ongoing. Data from this study cannot be shared because of information governance restrictions in place to protect confidentiality. Access to data can be requested via application to the Healthcare Quality Improvement Partnership (www.hqip.org.uk/national-programmes/accessing-ncapop-data/).

## Supplementary material

For supplementary material accompanying this paper visit http://dx.doi.org/10.1192/bjo.2021.1021. click here to view supplementary material
