# Supplementary figures and images for: Suicide rates among patients subject to community treatment orders in England during 2009–2018
Source: BJPsych Open. 2021 Oct 1;7(6):e180. doi: 10.1192/bjo.2021.1021 (PMC8503909; doi:10.1192/bjo.2021.1021)

**Supplementary data**

**Fig 1: Rates of suicide in CTO patients and in all discharged patients**


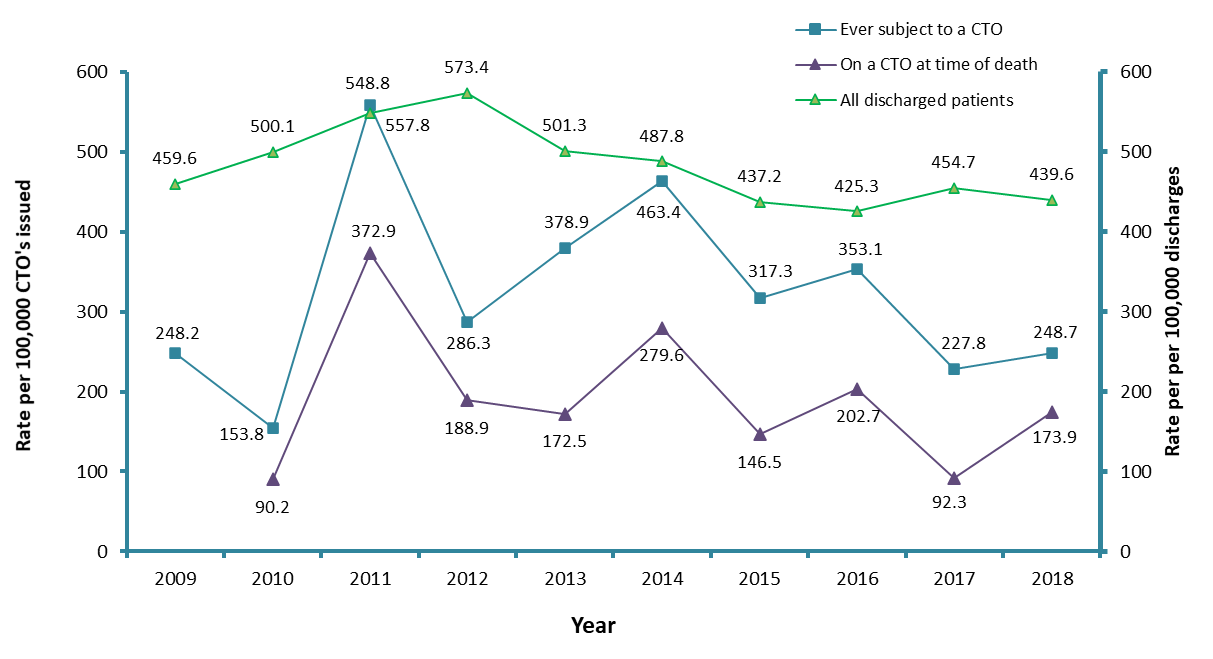

Supplement: Supplementary file 1 [file S2056472421010218sup001.docx]
